# Probable neurosyphilis-associated longitudinally extensive transverse myelitis with severe bladder dysfunction and radiologic resolution: a case report

**DOI:** 10.1186/s12883-026-05273-x

**Published:** 2026-08-18

**Authors:** Chih-Hsiang Wang, Yu-Cheng Chu

**Affiliations:** 1 Neurocritical Care Unit, Department of Critical Care Medicine, Far Eastern Memorial Hospital, New Taipei City, Taiwan (R.O.C.); 2 Department of Neurology, Far Eastern Memorial Hospital, New Taipei City, Taiwan (R.O.C.); 3 Graduate Institute of Management, Chang Gung University, Taoyuan City, Taiwan (R.O.C.)

**Keywords:** Neurosyphilis, Longitudinally extensive transverse myelitis, Syphilitic myelitis, Spinal cord, Bladder dysfunction, AQP4, MOG, Case report

## Abstract

**Background:**

Longitudinally extensive transverse myelitis (LETM) is often investigated as an autoimmune inflammatory myelopathy, but treatable infectious mimics should also be considered. Neurosyphilis-associated myelitis is uncommon and may resemble aquaporin-4 or myelin oligodendrocyte glycoprotein antibody-associated disease.

**Case presentation:**

A 31-year-old man presented with a one-month history of progressive bilateral lower-limb thermal dysesthesia and hyperesthesia, proximal leg weakness, pyramidal signs, urinary retention, and fecal incontinence. Spinal magnetic resonance imaging showed a longitudinally extensive central thoracic cord T2 hyperintensity from T6 to T10. Serum syphilis serology and cerebrospinal fluid Venereal Disease Research Laboratory testing were reactive, whereas human immunodeficiency virus testing was negative, serum aquaporin-4 and serum myelin oligodendrocyte glycoprotein antibodies were negative, and oligoclonal bands were absent. Because the initial cerebrospinal fluid sample was blood-contaminated and showed no pleocytosis, the diagnosis was considered probable neurosyphilis-associated LETM rather than definitive syphilitic myelitis. The patient received ceftriaxone-based anti-treponemal therapy with adjunctive corticosteroids. Follow-up cerebrospinal fluid Venereal Disease Research Laboratory testing became non-reactive, spontaneous voiding recovered, and follow-up spinal magnetic resonance imaging showed radiologic resolution.

**Conclusions:**

Probable neurosyphilis-associated LETM can mimic seronegative inflammatory myelitis. Syphilis testing should be considered early in LETM, particularly when prominent sphincter dysfunction is present. Diagnostic certainty may be limited by atypical cerebrospinal fluid findings, blood contamination, and concomitant antimicrobial and corticosteroid treatment.

**Supplementary Information:**

The online version contains supplementary material available at https://doi.org/10.1186/s12883-026-05273-x.

## Background

Longitudinally extensive transverse myelitis (LETM) is a radioclinical syndrome with a broad differential diagnosis, including autoimmune, infectious, vascular, neoplastic, metabolic, and paraneoplastic disorders. In contemporary neurological practice, LETM often raises concern for aquaporin-4 (AQP4)-related neuromyelitis optica spectrum disorder (NMOSD) or myelin oligodendrocyte glycoprotein (MOG) antibody-associated disease. However, infectious etiologies remain clinically important because some are potentially reversible if identified early.

Neurosyphilis is a classic neurological mimic. Central nervous system involvement can occur at any stage of syphilis, yet spinal cord involvement is uncommon. Syphilitic myelitis may present with sensory disturbance, paraparesis, pyramidal signs, and sphincter dysfunction, together with longitudinal spinal cord abnormalities on magnetic resonance imaging (MRI). Because these features overlap with autoimmune inflammatory myelitis, diagnosis may be challenging, particularly in human immunodeficiency virus (HIV)-negative patients without other overt infectious manifestations. Current guidance emphasizes integration of neurological findings, serum serology, cerebrospinal fluid (CSF) findings, and treatment response rather than reliance on a single test [1, 2].

We report a young HIV-negative man with probable neurosyphilis-associated LETM who presented with progressive thoracic myelopathy and marked urinary dysfunction. The case is clinically instructive because it resembled seronegative inflammatory myelitis, was complicated by blood-contaminated CSF and absence of CSF pleocytosis, and showed favorable longitudinal recovery with radiologic resolution after treatment.

## Case presentation

**Fig. 1:**
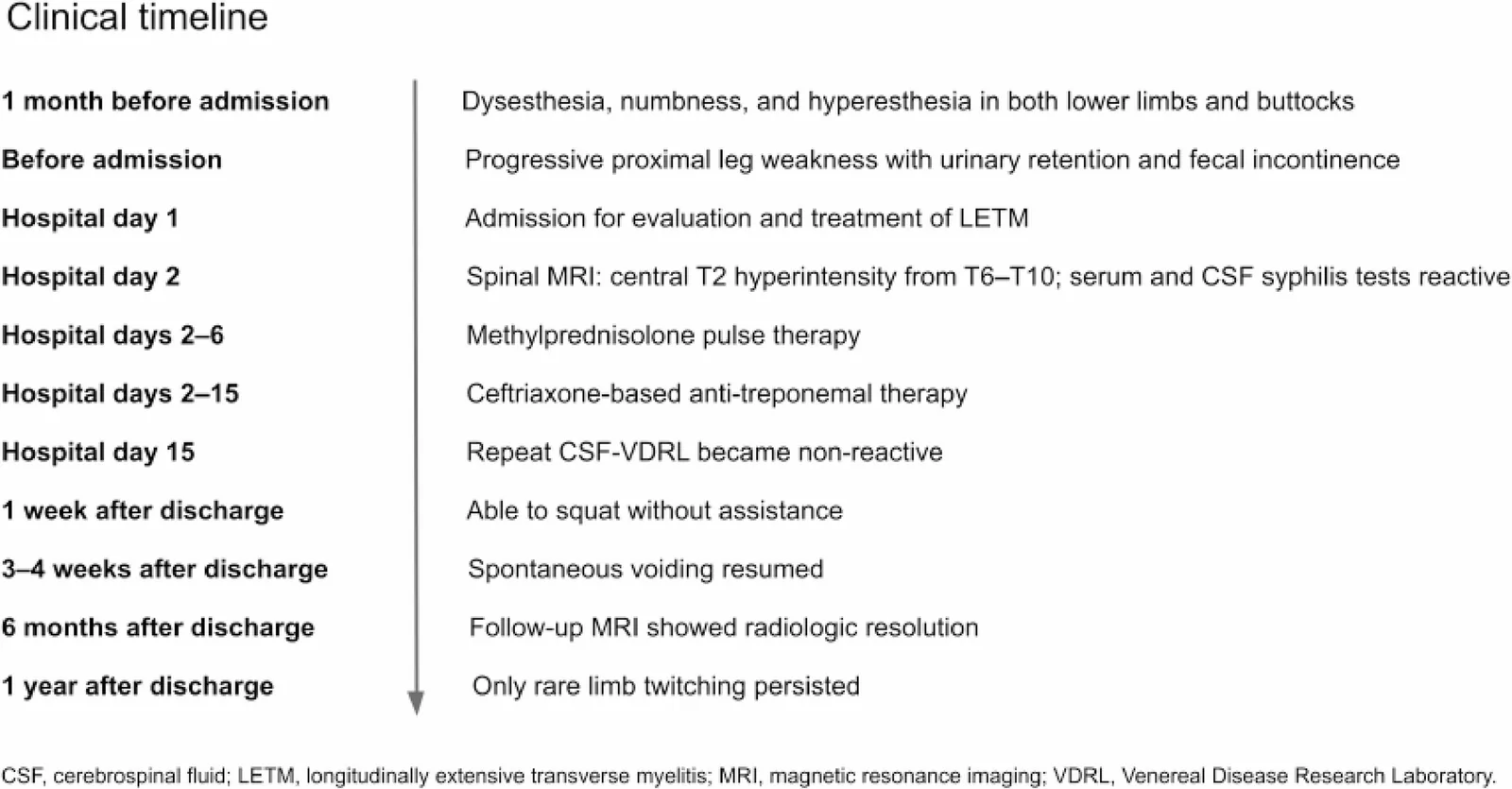
Clinical timeline. The timeline summarizes the sequence of symptom evolution, diagnostic work-up, treatment, and longitudinal recovery. CSF, cerebrospinal fluid; LETM, longitudinally extensive transverse myelitis; MRI, magnetic resonance imaging; VDRL, Venereal Disease Research Laboratory

A 31-year-old man without known systemic disease presented with a one-month history of progressive heat sensation, intermittent numbness, and hyperesthesia involving both lower limbs and the bilateral buttocks. The sensory symptoms gradually worsened and extended proximally. He subsequently developed difficulty climbing stairs, urinary retention requiring Foley catheterization, and fecal incontinence. He had initially sought medical attention at another hospital, where spinal imaging was reportedly interpreted as showing no definite myelopathy. Because symptoms persisted and progressed, he presented to our neurology service for further evaluation. He reported epidemiologically relevant sexual exposure for syphilis. No prior painless genital ulcer, mucocutaneous rash, or previous diagnosis or treatment of syphilis was documented in the available clinical record. The clinical course is summarized in Fig. 1.

On admission, he was alert and fully oriented. Cranial nerve examination was unremarkable, with full extraocular movements and no facial weakness. Motor examination showed preserved strength in both upper extremities, whereas proximal strength in both lower extremities was decreased to Medical Research Council grade 4/5. Sensory examination showed thermal dysesthesia and hyperesthesia involving both lower limbs and buttocks, without a clearly documented objective sensory level or modality-specific sensory loss. Deep tendon reflexes were brisk in both lower extremities, with bilateral Babinski signs and ankle clonus. The overall clinical picture suggested thoracic myelopathy with upper motor neuron involvement and significant sphincter dysfunction.

**Fig. 2:**
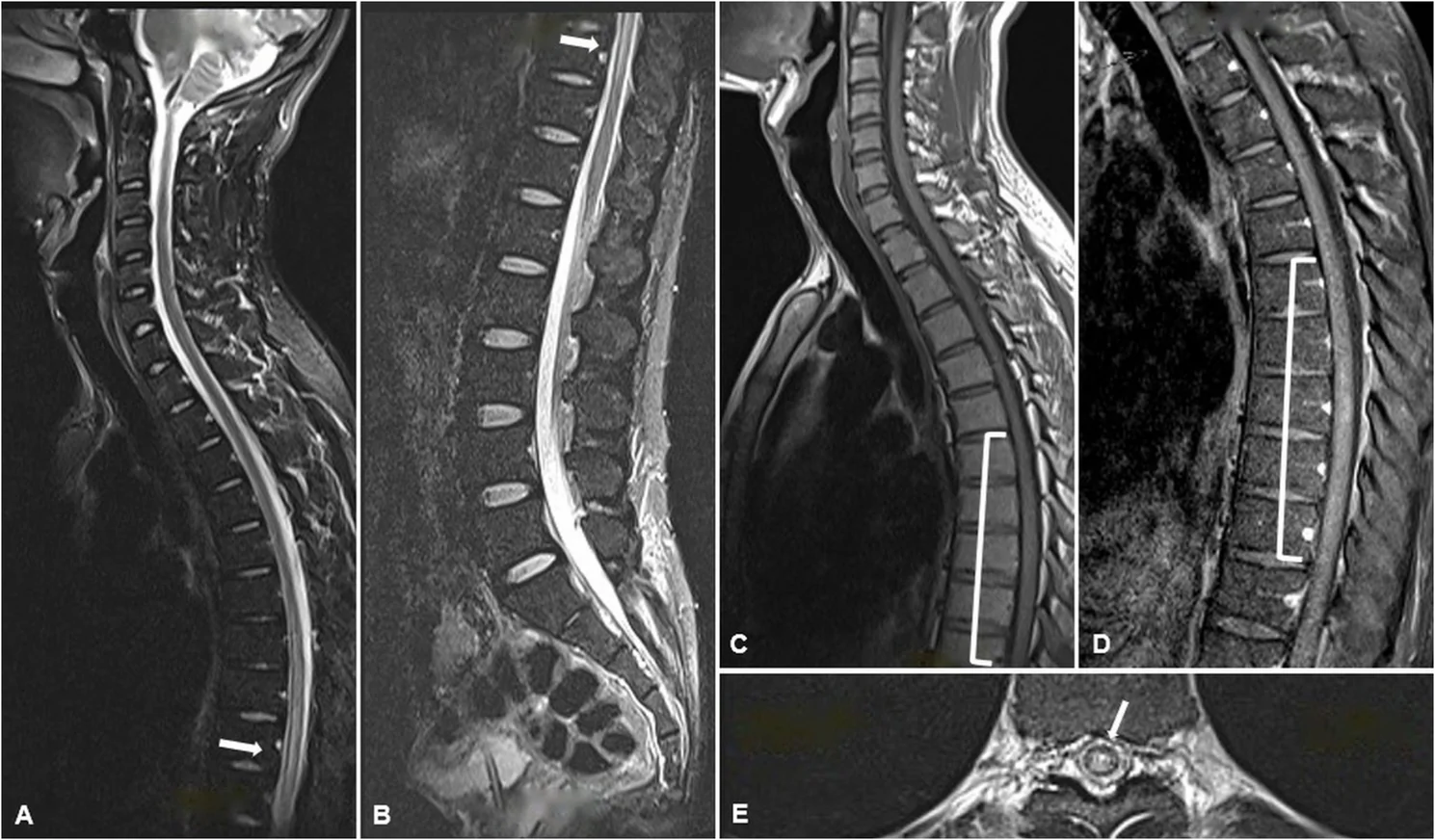
Initial spinal MRI demonstrating thoracic longitudinally extensive transverse myelitis. Initial spinal magnetic resonance imaging showed longitudinally extensive central thoracic cord T2 hyperintensity from T6 to T10. **A**, **B** Sagittal T2-weighted/STIR images showed central cord hyperintensity at the involved thoracic levels. **C**, **D** Corresponding T1-weighted and post-contrast T1-weighted images are shown. **E** Axial T2-weighted image at the involved thoracic level demonstrated central intramedullary hyperintensity. LETM, longitudinally extensive transverse myelitis

Whole-spine MRI demonstrated a longitudinally extensive central thoracic cord T2 hyperintensity from T6 to T10 (Fig. 2). Brain MRI showed no remarkable abnormality. At that stage, the differential diagnosis included idiopathic transverse myelitis, autoimmune myelitis, and NMOSD. Subsequent serum testing showed negative AQP4-IgG and negative MOG-IgG using the IIFT-03 NMOSD Screen, a cell-based indirect immunofluorescence screening assay. Both antibodies were tested in serum only; CSF AQP4-IgG and CSF MOG-IgG were not tested.

Further investigation disclosed strongly positive syphilis serology. Serum rapid plasma reagin (RPR) was reactive at 1:128, and Treponema pallidum particle agglutination (TPPA) assay was positive at 1:20480. CSF Venereal Disease Research Laboratory (VDRL) testing was reactive at 1:8. HIV antigen/antibody testing was negative. The initial CSF sample showed 3840 red blood cells/microL and 2 white blood cells/microL, without inflammatory pleocytosis. CSF treponemal testing, such as CSF fluorescent treponemal antibody absorption (FTA-ABS) or CSF TPPA/TPHA, was not performed. The CSF IgG index was mildly elevated during admission and later normalized. Infectious disease consultation favored neurosyphilis with associated myelitis.

Electrophysiological studies were not suggestive of peripheral neuropathy or dorsal column conduction impairment. Nerve conduction studies were within normal limits, and somatosensory evoked potentials were normal. Visual evoked potentials were also unremarkable. Because of persistent urinary retention, urological evaluation was arranged, and subsequent urodynamic study demonstrated an acontractile bladder with urinary retention.

During hospitalization, the patient received ceftriaxone 2 g every 12 h for 14 days after infectious disease consultation. The regimen was selected because intravenous aqueous penicillin G was not locally available at the time. Because inflammatory myelitis remained an important early consideration, high-dose methylprednisolone pulse therapy was administered for five days, followed by oral prednisolone tapering. Fecal incontinence improved after pulse therapy, although initial Foley catheter removal was unsuccessful. Repeat CSF examination after completion of treatment showed no pleocytosis, normalized protein parameters, and non-reactive CSF-VDRL. He was discharged with outpatient follow-up in neurology, urology, and infectious disease clinics.

**Fig. 3:**
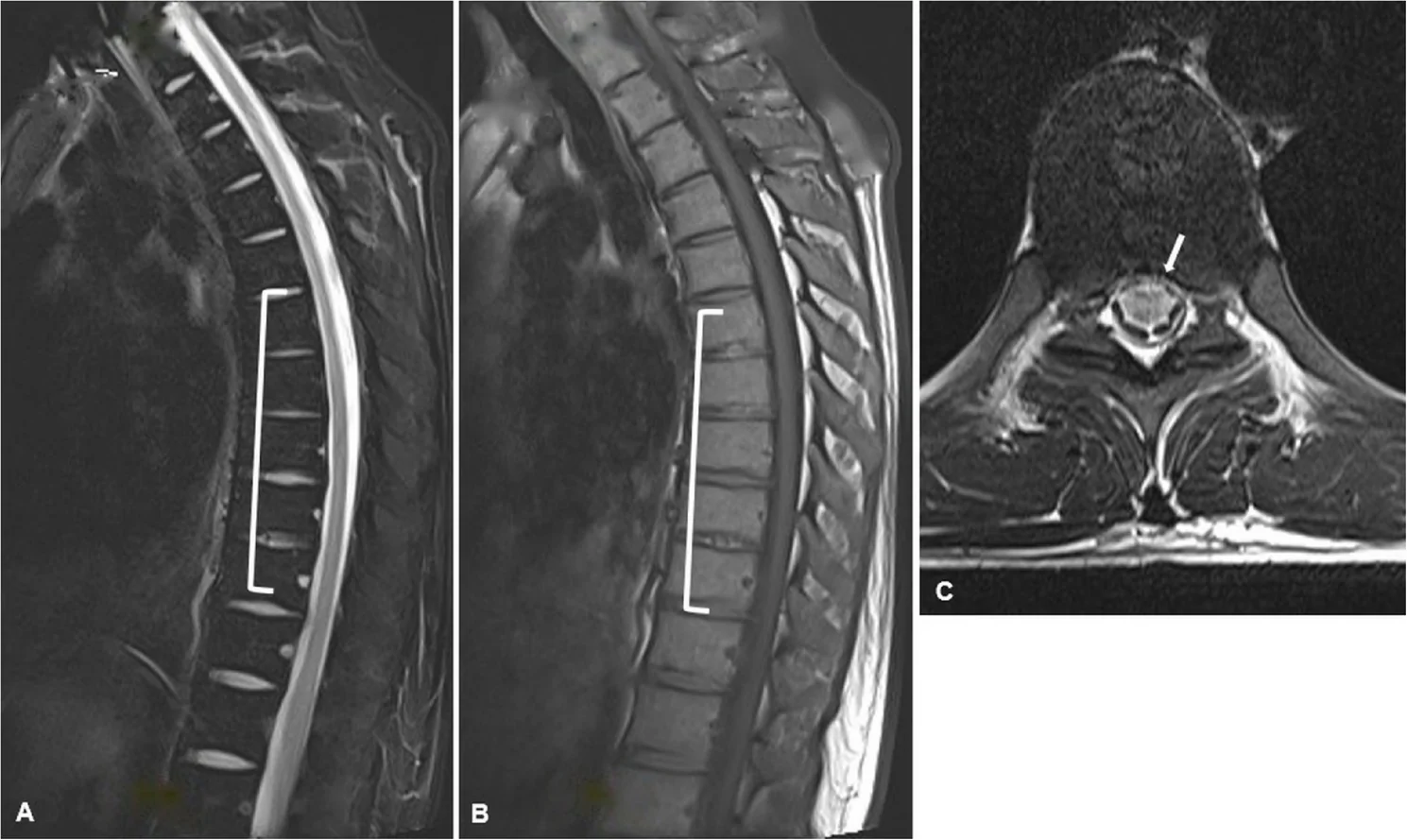
Follow-up spinal MRI showing radiologic resolution. Follow-up spinal magnetic resonance imaging obtained 6 months after treatment demonstrated interval resolution of the previously observed thoracic cord T2 signal abnormality. **A** Sagittal T2-weighted/STIR image at the corresponding thoracic levels. **B** Corresponding sagittal T1-weighted image. **C** Axial T2-weighted image at the corresponding thoracic level

During outpatient follow-up, neurological function improved gradually. One week after discharge, he was able to squat without assistance and planned Foley catheter removal soon thereafter. Approximately three and a half weeks after discharge, spontaneous voiding had recovered. Follow-up spinal cord MRI obtained six months after treatment demonstrated interval resolution of the previously observed thoracic cord signal abnormality (Fig. 3). At one year of follow-up, only rare limb twitching episodes remained.

### Patient perspective

A formal patient perspective on the treatment course was not separately obtained beyond written informed consent for publication of the de-identified clinical details and imaging data.

## Discussion and conclusions

This case illustrates probable neurosyphilis-associated LETM as a treatable diagnostic consideration in a patient initially resembling AQP4/MOG-seronegative inflammatory myelitis. The diagnosis was supported by compatible thoracic myelopathy, prominent sphincter dysfunction, reactive serum and CSF syphilis testing, exclusion of common autoimmune demyelinating etiologies, CSF-VDRL conversion to non-reactive status, recovery of spontaneous voiding, and radiologic resolution. However, because the initial CSF was blood-contaminated and showed no pleocytosis, the diagnostic picture should be interpreted as suggestive rather than fully conclusive.

The CSF findings are central to this uncertainty. Although a reactive CSF-VDRL has high specificity for neurosyphilis in the appropriate setting, current guidance considers it diagnostic only when the specimen is not blood-contaminated [1, 2]. In this patient, the initial CSF was substantially blood-contaminated and showed no pleocytosis. The absence of CSF pleocytosis is atypical for active neurosyphilitic meningomyelitis and weakens diagnostic certainty. Additional CSF treponemal testing, such as CSF FTA-ABS or CSF TPPA/TPHA, might have provided useful supportive information. A negative CSF treponemal test would have made neurosyphilis less likely, whereas a positive result would have supported the diagnosis but would still not have definitively established active neurosyphilitic myelitis in the setting of reactive serum treponemal serology and blood-contaminated CSF [1]. The reported sexual exposure provided relevant epidemiological context, but the timing of syphilis acquisition could not be determined from the record. No prior episode suggestive of primary or secondary syphilis was documented, although neurosyphilis can occur at any stage of infection [1, 2].

Other causes of LETM were considered. Vascular myelopathy was less favored because the clinical course was subacute and progressive over approximately one month rather than hyperacute or stepwise. Spinal cord infarction was therefore less consistent with the temporal profile. A spinal dural arteriovenous fistula was also considered less likely because the available MRI report did not describe prominent perimedullary flow voids or a typical progressive congestive myelopathy pattern. Other infectious causes of myelitis were considered less likely because the patient was afebrile, HIV testing was negative, and CSF pleocytosis was absent. Systemic inflammatory or connective-tissue disease-associated myelitis was not supported by the available clinical record, although rare inflammatory, paraneoplastic, and metabolic causes could not be completely excluded in a single retrospective case report.

The pathophysiology of syphilitic myelitis is incompletely understood and may involve more than one mechanism. Reported mechanisms by which Treponema pallidum affects the spinal cord include syphilitic meningomyelitis with meningeal and perivascular inflammatory infiltration, meningovascular involvement with obliterative endarteritis or vasculitis, and secondary ischemic injury of the spinal cord [3–8]. These processes may coexist and may explain the variable MRI patterns reported in syphilitic myelitis, including longitudinal T2 hyperintensity, contrast enhancement, and occasional radiologic improvement after treatment [3–8]. In the present case, the absence of tissue confirmation and the non-specific MRI pattern preclude attribution to a single mechanism.

The treatment course also reflects the real-world overlap between infectious and inflammatory spinal cord syndromes. Current guidance recommends intravenous aqueous penicillin G as the preferred regimen for neurosyphilis, whereas ceftriaxone is supported only as an alternative regimen with limited data [2]. In this case, ceftriaxone 2 g every 12 h was selected after infectious disease consultation because intravenous aqueous penicillin G was not locally available. The twice-daily dosing reflected local practice for suspected central nervous system infection rather than a regimen specifically validated for neurosyphilitic myelitis. In addition, corticosteroids were administered because inflammatory myelitis remained a competing diagnosis in the acute phase. Therefore, clinical improvement cannot be attributed to any single intervention, and this case should not be interpreted as evidence that the ceftriaxone regimen used here is superior to guideline-recommended therapy.

The added value of this case is the longitudinal documentation of a probable neurosyphilis-associated LETM phenotype with severe bladder dysfunction, recovery of spontaneous voiding, conversion of CSF-VDRL to non-reactive status, and radiologic resolution. These findings support inclusion of syphilis testing early in the evaluation of LETM, especially when prominent sphincter dysfunction is present and AQP4/MOG antibodies are negative. However, several limitations should temper interpretation: the initial CSF sample was blood-contaminated, CSF pleocytosis was absent, CSF treponemal testing was not performed, AQP4-IgG and MOG-IgG testing was limited to serum, the ceftriaxone regimen was not a standard guideline regimen, and corticosteroids were administered concurrently. Thus, the case supports neurosyphilis as an important treatable diagnostic consideration in LETM, but it does not conclusively prove the mechanism of myelitis or the independent effect of any single treatment.

## Supplementary Information

Supplementary Material 1.

## Data Availability

The data supporting the findings of this case report are included within the article and its figures. Additional individual-level clinical data are not publicly available due to patient privacy and ethical restrictions. Further inquiries may be directed to the corresponding author and will be considered subject to institutional policies and applicable ethical approvals.

## Abbreviations

AQP4: Aquaporin-4
CSF: Cerebrospinal fluid
FTA-ABS: Fluorescent treponemal antibody absorption
HIV: Human immunodeficiency virus
LETM: Longitudinally extensive transverse myelitis
MOG: Myelin oligodendrocyte glycoprotein
MRI: Magnetic resonance imaging
NMOSD: Neuromyelitis optica spectrum disorder
RPR: Rapid plasma reagin
STIR: Short tau inversion recovery
TPHA: Treponema pallidum haemagglutination assay
TPPA: Treponema pallidum particle agglutination
VDRL: Venereal Disease Research Laboratory
